# Scaling of domain cascades in stripe and skyrmion phases

**DOI:** 10.1038/s41467-019-09934-z

**Published:** 2019-04-30

**Authors:** A. Singh, J. C. T Lee, K. E. Avila, Y. Chen, S. A. Montoya, E. E. Fullerton, P. Fischer, K. A. Dahmen, S. D. Kevan, M. K. Sanyal, S. Roy

**Affiliations:** 1 Saha Institute of Nuclear Physics, HBNI, 1/AF Bidhannagar, Kolkata, West Bengal 700064 India; 2 Materials Sciences Division, Lawrence Berkeley National Lab, Berkeley, CA 94720 USA; 3 Advanced Light Source, Lawrence Berkeley National Lab, Berkeley, CA 94720 USA; 4 Department of Physics, University of Illinois Urbana-Champaign, Urbana, IL 61801 USA; 5 Center for Memory and Recording Research, University of California San Diego, La Jolla, CA 92093 USA; 6 Department of Physics, University of California, Santa Cruz, CA 95064 USA

**Keywords:** Magnetic properties and materials, Phase transitions and critical phenomena, Spintronics

## Abstract

The origin of deterministic macroscopic properties often lies in microscopic stochastic motion. Magnetic fluctuations that manifest as domain avalanches and chaotic magnetization jumps exemplify such stochastic motion and have been studied in great detail. Here we report Fourier space studies of avalanches in a system exhibiting competing magnetic stripe and skyrmion phase using a soft X-ray speckle metrology technique. We demonstrate the existence of phase boundaries and underlying critical points in the stripe and skyrmion phases. We found that distinct scaling and universality classes are associated with these domain topologies. The magnitude and frequency of abrupt magnetic domain jumps observed in the stripe phase are dramatically reduced in the skyrmion phase. Our results provide an incisive way to probe and understand phase stability in systems exhibiting complex spin topologies.

## Abstract (web summary)

Switching of magnetic materials often occurs through discrete random avalanches. Singh et al. observe sharply reduced avalanches in the topologically protected skyrmion phase of a Fe/Gd heterostructure and obtain different critical behaviour in the stripe and skyrmion phases, suggesting distinct universality classes.

## Introduction

Nanoscale fluctuations and stochastic motion of the atomic and/or electronic constituents have profound impact on the emergence of functionality in complex materials^1^. Fluctuations are of particular scientific interest in quantum materials where correlation effects and competing interactions yield a symmetry breaking non-trivial ordering phenomena, such as stripe order, spin and orbital order, or in topological spin phases that give rise to e.g. chiral spin textures^2–6^. Recent numerical results show importance and connection between fluctuating stripe phases and mechanism of high *T*_C_ superconductivity^4,7^. Skyrmions and quantum topological spin texture are other class of examples, where theoretical calculations predict the important role of spin fluctuations in stabilizing the topological phase^8^.

Spin, charge, and orbital motions can be scattered by fluctuations, thereby generating entropy and losing information. Controlling and minimizing this decoherence will be a key feature of deploying quantum matter in emerging information technologies. An interesting example will be a lattice of skyrmions. Due to the topological protection and existence of multiple length scale energetics, skyrmions scatter weakly from defects and can be moved relatively easily through the lattice. A key question is does the topological protection that exists for single skyrmion extends to the skyrmion lattice that contains domain walls? How are skyrmion domain wall fluctuations affected by an applied field excursion? It is conceivable that heterogeneity in order and morphology is associated with intermittent events at the nanoscale that spawn statistically self-similar spatial and/or temporal structures^9^.

Recently discovered skyrmions provide an ideal platform to study how topology affects fluctuations and scaling. Skyrmions are relevant in various condensed matter systems ranging from 2D quantum Hall systems^10,11^, liquid crystals^12^, multiferroics^13^, ferroelectrics^14^ to even Bose condensates^15^. Specifically, skyrmions in magnetic systems provide unusual flexibility in measuring the response of nanoscale topological features under applied fields^6,16–18^. Skyrmions are particle-like chiral magnetic spin structures that can get arranged in a hexagonal lattice. The topology of a skyrmion is described in terms of a winding-number, which is a quantized and conserved quantity. This provides topological protection from defects and pinning sites, and makes skyrmions a potential candidate for low power memory and logic applications^18,19^. Since device stability depends crucially on fluctuations, it is important to understand the effect of topology on the abrupt domain jumps and avalanches, for example, as the applied magnetic field is varied. In spite of a lot of progress in materials discovery for skyrmions, questions about stability, abrupt jumps, and domain stability have not been addressed in detail. There are very limited studies focused on this topic, for example, in a recent theoretical study, it has been predicted that skyrmions can exhibit avalanches with power law distribution and universality has been discussed^20^. Topologically trivial magnetic structures, such as bubble domains, have been discussed in the context of cascades and scaling behavior where, under applied magnetic field the bubble domains were found to self-organize in a sub-critical state^21^.

It is known that Fe/Gd heterostructure is a ferrimagnet and can be made to exhibit perpendicular magnetic anisotropy by suitably choosing the thickness and composition^22,23^. Depending on temperature and applied field, the Fe/Gd heterostructure exhibits three distinct phases: (i) an ordered stripe phase, (ii) a disordered stripe phase, and (iii) a skyrmion lattice phase^24^. The existence of the three phases with distinct phase boundaries in Fe/Gd thin film has remarkable similarity with physical systems that display two-dimensional modulated structures^25^. For example, monolayers of rare gas adsorbed on graphite show three phases: a disordered fluid-like phase, an ordered phase registered with the underlying lattice, and an incommensurate phase. The three phase-lines meet at a multi-critical point near which enhanced fluctuations are observed. Similar phase diagram also exists for Ising systems where competing interaction gives rise to charge or spin density waves^26^. Theoretically predicted phase diagram for such systems is extremely rich, with a number of transitions between periodic phases, including a floating unpinned phase. It is further shown that entropy contributions and fluctuations determine stability of these different phases. It is conceivable that the multiple phase-lines in the Fe/Gd system may also have an underlying critical point.

Central to the understanding of fluctuation and critical behavior is the determination of existence of power law behavior. Power law dependencies and the absence of characteristic scales are of utmost importance in understanding the emerging macroscopic properties from microscopic stochastic phenomena^27–33^. The power laws are affected by different parameters such as, material microstructure, external stress, demagnetizing field, etc. If power laws obtained at different conditions can be collapsed to a unique scaling behavior, the system is said to exhibit universality. Scaling and universality implies that the relevant symmetries, interactions, and conservation laws influence the behavior of the system, while many other quantitative details are irrelevant.

**Fig. 1:**
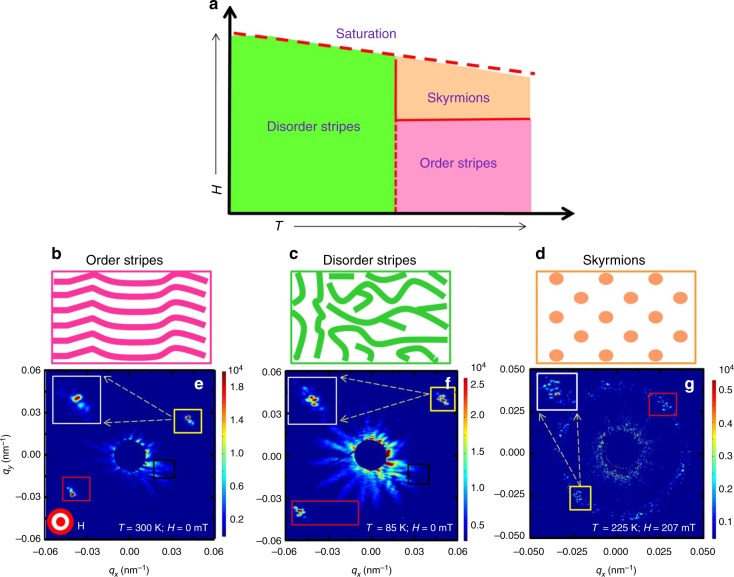
Magnetic diffraction from stripes and skyrmions. **a** Schematic of phase diagram of the Fe/Gd sample as a function of temperature and applied magnetic field. **b**–**d** Schematic of order stripes, disorder stripes, and skyrmion phase. **e**–**g** Magnetic diffraction spots in the three phases. The insets show enlarged image of the magnetic diffraction spots that are enclosed in yellow box. The red and black boxes are the regions of interest of magnetic diffraction and airy fringes, respectively, for calculating the pair-wise correlation coefficients. The box sizes are the same for each data set, except for the disorder stripe diffraction spot where it is elongated to account for the peak broadening

Here we show that resonantly tuned coherent X-ray scattering provides a unique platform to perform element-specific studies of stochastic events to quantitatively characterize fluctuations. We studied a Fe/Gd thin film heterostructure that exhibit highly tunable stripe and skyrmion phases (schematic of the phase diagram is shown in Fig. 1a) and we addressed the question of phase boundaries and underlying critical point in the context of stripe and skyrmion phases. We show that even within a stripe phase an order and disorder phase is separated by critical points. In the pure skyrmions phase, magnitude of domain cascades are dramatically reduced, however, the distribution of the cascade size follow similar pattern as in stripe phase. Interestingly, the divergence of correlation length is much faster for the stripes near the critical point than for the skyrmions, which would imply that skyrmions have a higher degree of criticalness. By analyzing the data within the framework of statistical mechanics we show here that the distribution of fluctuations could be collapsed to a unifying scale with parameters that are distinct in the stripes and skyrmions phases indicative of two separate universality classes.

## Results

### Resonant coherent soft X-ray scattering of stripe and skyrmion phases

Resonant coherent soft X-ray magnetic scattering patterns of the Fe/Gd multilayer are shown in Fig. 1e–g. Both stripe and skyrmion peaks are modulated with speckles that indicate finite-sized domain formation. We used the following field protocol consistently throughout the experiment to get to the specific phases. The sample was subjected to a magnetic field that was first raised to 500 mT, then reversed to −500 mT and finally reduced to zero before taking the measurements. The field ramp rate for the first two segments was 13 mT/s while the final drop of field from −500 to 0 mT to took place at a rate of 380 mT/s. We started our measurement at this zero-field condition and proceeded to measure diffraction data as a function of applied magnetic field using a ramp rate of 1.575 mT/s. The above protocol was repeated at different temperatures between 85 and 300 K.

At room temperature an ordered stripe domain with periodicity of 138 ± 5 nm forms at zero field. Around 200 mT new peaks in the form of a distorted hexagonal pattern start to appear due to the onset of skyrmion formation. The periodicity of skyrmion hexagonal-lattice at 200 mT is 190 ± 5 nm. The ordering of the stripe was found to decrease as we lowered the temperature. At the lowest measured temperature of 85 K, we start with a disordered phase at zero-field condition. As the field is increased the diffraction spots smear into arcs indicative of formation of meandering or disordered stripes. The amount of field-dependent rotation of the diffraction spots decreases with increasing temperature, and stops eventually at some higher temperature where hexagonal Bragg spots appear due to the formation of skyrmions. Consequentially, there must exist a minimum temperature where there is a field-driven transition from stripe to skyrmion phase, which we have identified as *T* = 205 K. The rotation and movement of the diffraction pattern gives a hint about the presence of temperature and applied field-dependent instability in the system.

**Fig. 2:**
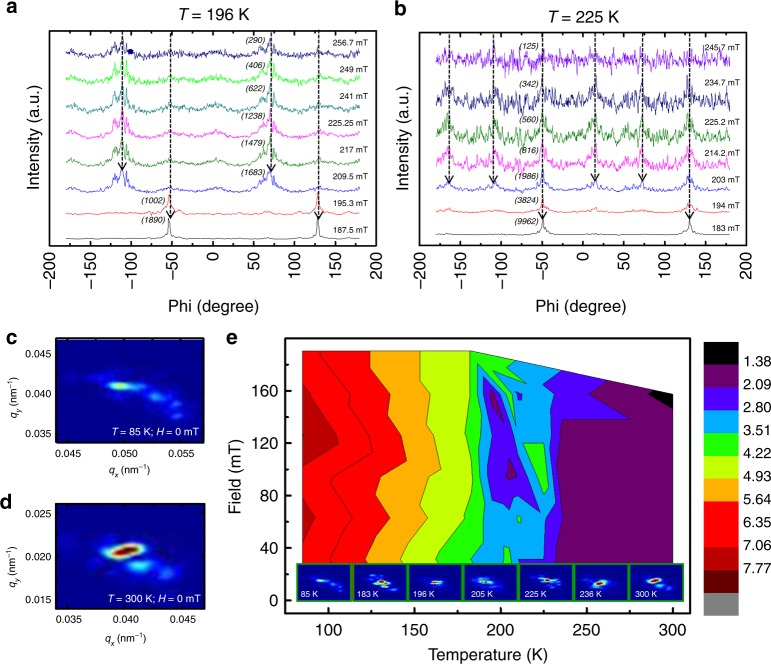
Evolution of stripe and skyrmion phase: Azimuthal variation of scattered intensity for different field values at (**a**) 196 K and (**b**) 225 K. The black dotted arrows show the position of stripe and skyrmion peaks. **c**, **d** Enlarged image of the diffraction spot for disordered stripes and ordered stripes, respectively. **e** Contour plot of angular full width half maximum (FWHM) of the stripe diffraction spot along the azimuthal direction as a function of field and temperature. Color bar represents the FWHM of the diffraction spot. The FWHM is related to degree of disorder of the stripe domains. The panel inset shows representative diffraction pattern as the temperature is changed. Clear evolution from a broad pattern to a strong diffraction peak is evident

To illustrate how the structure evolves from stripe to skyrmion phase, we plot in Fig. 2a, b line scans of the diffraction data along the azimuthal direction (constant scattering momentum contour) as a function of applied magnetic field. At *T* = 196 K (near the stripe–skyrmion phase boundary), we initially obtain two diffraction spots corresponding to an ordered stripe state. The two spots persist to around 200 mT when two additional new spots appear 50° in azimuthal angle away from the original spot position. The intensity of the original spot diminishes although it does not disappear. In effect this means the stripes predominantly rotate while some stripes persist in the original direction. In contrast, the skyrmion phase at *T* = 226 K (Fig. 2b) starts with two diffraction spots corresponding to stripes and develops as field increases into six spots characteristic of the skyrmion phase. Thus, as skyrmions form, four diffraction spots appear in addition to the two diffraction spots due to the stripes without involving any further rotations.

In Fig. 2c, d we show that diffraction peaks at zero field are much more spread out in the azimuthal direction in reciprocal space at 85 K compared to 300 K, indicating a shorter correlation length and hence a larger degree of disorder in the stripes. A contour plot of the full width half maximum (FWHM) of the stripe diffraction peak as a function of applied field and temperature in Fig. 2e shows that the degree of disorder is highest at the lowest measured temperature and decreases continuously till 230 K after which the FWHM becomes constant. Around *T* = 230 K a transition from disordered to ordered stripe is observed. We therefore conclude that our Fe/Gd undergoes a transition from disordered to ordered stripe phase at (*T*_Cstripe_, *H*_Cstripe_) ≡ (230 K, 148 mT) and stripe-to-skyrmion transition at (*T*_Csk_, *H*_Csk_) ≡ (205 K, 220 mT). The importance of these transition temperatures and fields in context of criticality will be discussed below.

### Determination of stochastic domain jumps using speckle metrology

**Fig. 3:**
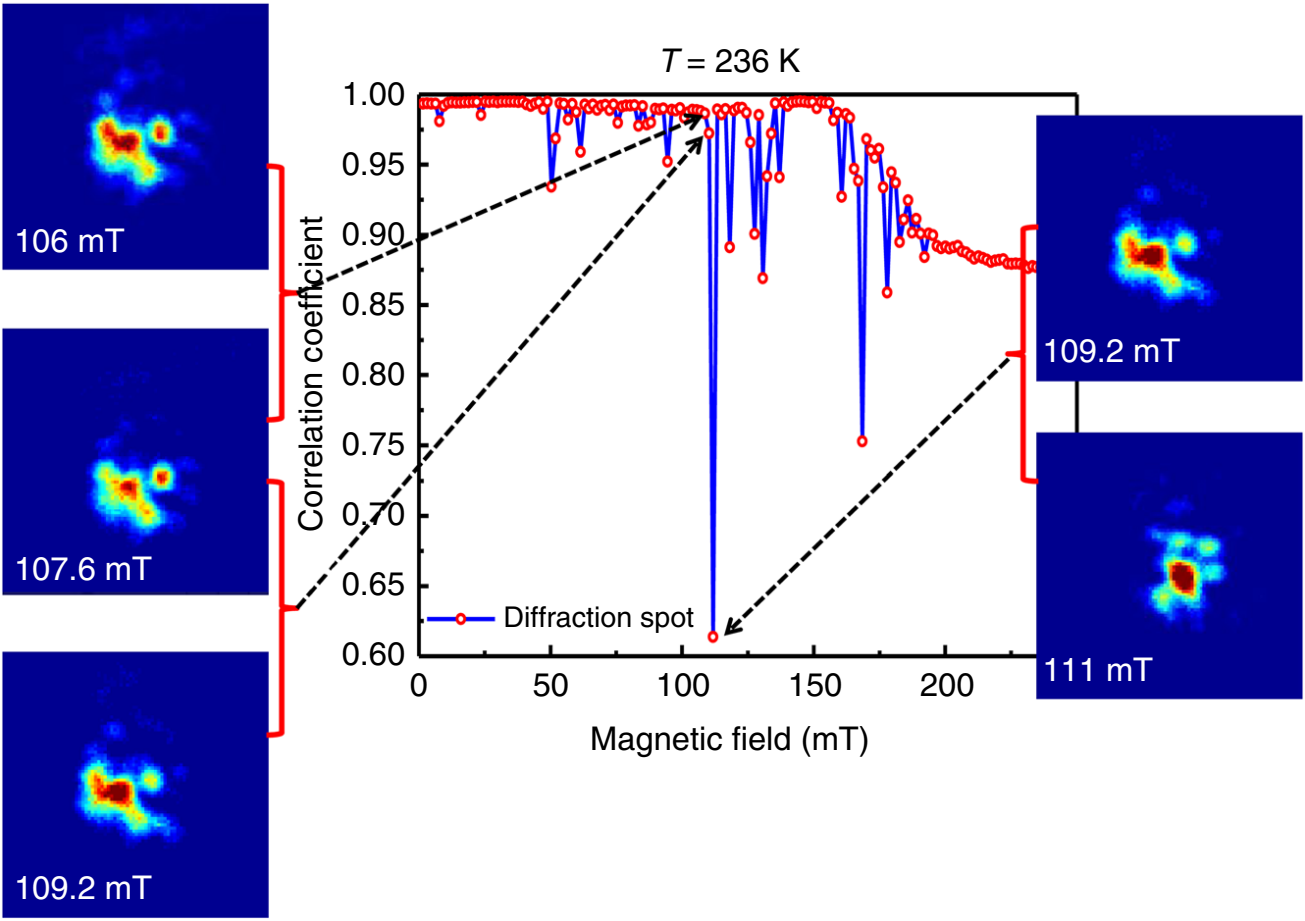
Pair-wise correlation coefficient: Pair-wise correlation function (blue line) versus perpendicular magnetic field. We show the speckle pattern at *T* = 236 K for four consecutive field values that produces small and big jumps. The speckle patterns are represented within an area of 90 by 90 pixels. Large change in speckle pattern gives rise to large magnitude of jump indicating a large change of domain morphology

Resonant coherent soft X-ray scattering from a magnetic sample gives rise to speckle patterns due to the interference of randomly phase-shifted waves that are scattered by the magnetic domains. A speckle pattern is a fingerprint, unique to the specific domain configuration illuminated by the X-ray beam. If the domain morphology changes either spontaneously or due to an external influence, then the speckle pattern will change as well. Such a change in an X-ray speckle pattern in reciprocal space provides a statistically significant measure of nanoscale changes in size, orientation and/or number density of the magnetic domains in real space. We developed a statistical measure of field-induced domain jumps with the normalized pairwise correlation coefficient, *p*, a standard tool to measure the similarity of data sets (see Supplementary Note 2). Specifically, we correlated consecutive speckle patterns, collected near the magnetic Bragg peaks discussed above, as the field was swept. If *p* = 1 for particular pairs of consecutive speckle patterns, then the domain patterns are the same and no jump has occurred. By contrast, *p* < 1 indicates a change in speckle pattern and domain morphology, and the magnitude of the drop of *p* from unity quantifies the magnitude of change in the domain morphology. This is shown graphically in Fig. 3 for a large domain jump with *p* = 0.61, for which the change in the speckle pattern is readily apparent.

The evolution of domain states under applied magnetic fields results through a collection of different physical mechanisms. If we were to perform pair correlations that take into account all potential field-dependent (e.g. stripe domain phase, coexisting stripe, and skyrmion phase) domain morphologies, then the resulting scaling laws would be complex and the dynamics from each phase could not be distinguished. (In Supplementary Figs. 2 and 3 we show simulation of jumps appearing due to position change and size change of domains.) Here, we performed analysis of pair correlations in distinct pure phases, namely stripe phases and the skyrmion phase using a well-established^34^ mean field model of avalanche dynamics. A magnetic material magnetizes in a phase through a series of jumps under slowly changing external field with no characteristic size scale, similar crackling happens in many systems when pushed slowly^9^. The statistical formalism used here has been making rapid progress to predict behavior of such systems on long scales of length and time, independent of many microscopic details.

**Fig. 4:**
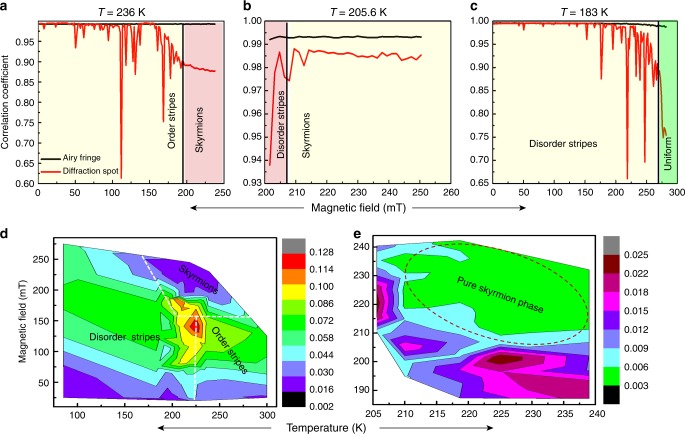
Average size of domain jump as a function of temperature and applied field: Plot of cross-correlation function (red line) versus magnetic field for (**a**) order stripe, (**b**) skyrmion, and (**c**) disorder stripe diffraction spots. The black line in the figures corresponds to the correlation value from non-magnetic region (airy fringes). Correlation between two consecutive image patterns was done for those regions as mentioned in Fig. 1 with the corresponding color boxes. The size and position of the boxes was maintained to be same for the entire field range. Color bar represents average jump size. Contour plot of average jumps size 〈*X*〉 as a function of field and temperature for (**d**) entire magnetic phase and (**e**) skyrmion phase only. A peak in the jump size in (**d**) (red area ≈ 225 K, 150 mT) suggests a critical point

Figure 4a–c shows the variation of the pairwise correlation coefficient of two consecutive datasets as a function of magnetic field in the ordered stripe, skyrmion, and disordered stripe phases, respectively. The correlation curve of stripe phase shows multiple sharp drops (jumps) indicating abrupt changes in the topological configuration of domains (red curve in Fig. 4a). The onset of the jump is the point at which the slope of the correlation curve changes from zero or positive to negative while the position of a jump is defined as the value of the magnetic field where the slope of the correlation curve sharply changes its sign from negative to positive. The correlation curves were repeated for more than 100 hysteresis loops at each temperature to build up statistics. The appearance of the abrupt jumps is reminiscent of domain avalanches/cascades observed in Barkhausen events^1,8,10^.

We obtained direct experimental evidence that the magnitude and frequency of jumps in the skyrmion phase is significantly smaller compared to those in the stripe phase (for example, compare magnitude of jumps in Fig. 4b with Fig. 4a–c). The reduced jump size is most likely a consequence of the stability due to topologically protective nature of the skyrmions. In the disordered stripe phase the size of the jumps are smaller at lower field values, but at higher applied field large jumps dominate (Fig. 4c). Interestingly, large and more cascades appear in the same field region where the skyrmions would appear for *T* ≥ 205 K. Since the characteristics of jumps are representative of a phase, a change in average jump size indicates a phase change and may have an underlying critical point.

A contour plot of the average jump size 〈*X*〉 as a function of field and temperature is a simple way to visualize the jump characteristics and ascertain probable presence of critical points. In Fig. 4d we show a contour plot spanning all the three magnetic phases present in the sample as a function of field and temperature. The contour plot shows that the average size of jumps increase with both temperature and field. By approaching from any direction in the contour plot it is observed that in the stripe phase, the average jump size shows a maximum value near the point *T* ∼ 225 K and *H* ∼ 148 mT, indicating the presence of a critical point in temperature and field around these values.

The green region inside the contour plot (Fig. 4e) shows the temperature-field conditions where all the six peaks are of same intensity thereby representing the pure skyrmion phase. The pure skyrmion phase is surrounded by bigger jumps indicating presence of mixed phases, which is in close agreement with the line scan shown in Fig. 4a. As shown before in the line scans (Fig. 4a–c), the magnitude of jumps is similar in both ordered and disordered stripes, however, the average jumps size in the skyrmion phase is almost an order-of-magnitude smaller than in the stripe phase.

### Scaling collapse of jump data

To test criticality, we make an ansatz assuming that there is a critical point where the moments of the avalanche size distribution get very large. If there is a critical point, i.e. a non-equilibrium phase transition, then we can use the mean field model for avalanche dynamics from ref. ^34^, which has been tested in several systems like earthquakes, nanocrystals, and granular materials^33^. According to the avalanche model, the distribution of jump sizes is expected to have the following general form:

1 $${Z\left( {X,T,H} \right) = X^{ - a}f_1\left( {X\left| {T - T_{\mathrm{C}}} \right|^{\frac{1}{n}_T},X\left| {H - H_{\mathrm{C}}} \right|^{\frac{1}{n}_H}} \right)\sim X^{ - a}f\left( {\frac{X}{{X_{{\mathrm{max}}}}}} \right)}$$

where *X* is the jump size* T*_C_, *H*_C_ are critical temperatures and fields, respectively, and, *n*_*T*_ and *n*_*H*_ are critical exponents, and *X*_max_ ∼ (*ξ*(*T − T*_C_, *H − H*_C_))^1/*συ*^ is the maximum (cutoff) jump size and *ξ*(*t*, *h*) is the correlation length that diverges at the critical point, with *t* ≡ |*T* − *T*_C_| → 0 and *h* ≡ |*H* − *H*_C_| → 0. The critical exponents *n*_*T*_ and *n*_*H*_ are related to how the correlation length grows as one approaches the critical point along the *T* or *H* direction. 1/*συ* is the fractal dimension of the jumps, and the universal scaling function *f*(*x*) is expected to decay exponentially as *f*(*x*) = *A*e^−*Bx*^, with *A* and *B* being non-universal constants.

According to Eq. (1), the distributions follow a power law up to the maximum jump size cut-off values given by $X_{T{\mathrm{max}}}\sim \left| {T - T_{\mathrm{C}}} \right|^{\frac{1}{{n_T}}}$ for *H* = *H*_C_ and $X_{{\mathrm{Hmax}}}\sim \left| {H - H_{\mathrm{C}}} \right|^{\frac{1}{{n_H}}}$ for *T* = *T*_C_.

Further, we can also obtain cumulative distribution function (CCDF) given by

2 $${{\mathrm{CCDF}}\left( {X,T,H} \right) = X^{ - \left( {a - 1} \right)}g_1\left( {\frac{X}{{X_{{\mathrm{Tmax}}}}},\frac{X}{{X_{{\mathrm{Hmax}}}}}} \right)\sim X^{ - (a - 1)}g\left( {\frac{X}{{X_{{\mathrm{max}}}}}} \right),}$$

where, $g\left( x \right)\sim x^{a - 1}\mathop {\smallint }\nolimits_x^\infty {\mathrm{{e}}}^{ - A\prime t}.t^{ - a}{\mathrm{{d}}}t$, with *A*′ being a non-universal constant.

From Eq. (1) we can derive the relation for the average size of the jumps, $\left\langle {X} \right\rangle (T) = {\int}_0^\infty X Z\left( {X,T} \right){\mathrm{{d}}}X$, which at *H* = *H*_C_ is given by $\left\langle {X} \right\rangle (T)\sim \left| {T - T_{\mathrm{C}}} \right|^{(a - 2)/n_T}$.

Analogously, for temperature *T* = *T*_C_ we obtain $\left\langle {X} \right\rangle (H) \sim \left| {H - H_{\mathrm{C}}} \right|^{(a - 2)/n_H}$.

**Fig. 5:**
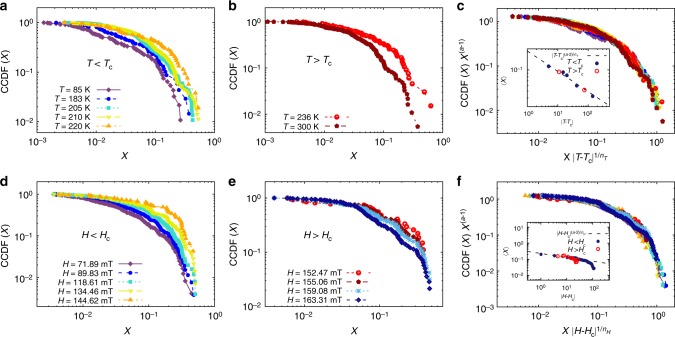
Scaling collapse for stripe phase: Stripe phase, with critical values *T*_Cstripe_ = 225 K and *H*_Cstripe_ = 148 mT. Complementary cumulative distribution function of domain cascades at (**a**, **b**) *H* = *H*_Cstripe_ and (**d**, **e**) *T* = *T*_Cstripe_. a, **d** Distribution corresponding to values below the critical point. **b** and **e** Distributions corresponding to values above the critical point. Scaling collapse at *H* = *H*_Cstripe_ (**c**) with critical exponents *a*_*H*stripe_ = 1.1 ± 0.3, *n*_*H*stripe_ = 3.73 ± 1.0 and *T* = *T*_Cstripe_ (**f**) with critical exponent values of *a*_*T*stripe_ = 1.1 ± 0.2, *n*_*T*stripe_ = 3.4 ± 0.6. Inset*:* The average size of the jumps as a function of the distance to the critical point, both below (blue circles) and above (red open circles) the critical transition. The dashed line represents the fit to 〈*X*〉(*μ*) ∼ |*μ* − *μ*_C_|^(*a*−2)/*n*^, with *μ* = *T* or *μ* = *H*

We have calculated the CCDF of the number of jumps (NOJ), i.e. CCDF = 1 − CDF and plotted it as function of Jump Size in log–log scale (details are given in the Supplemental Notes 4 and 5). Figure 5a, b show the CCDF for the stripe phase, at a fixed magnetic field *H*_Cstripe_, for temperatures below and above *T*_Cstripe_, respectively. Similarly, the CCDF at fixed temperature *T*_Cstripe_ for fields below and above *H*_Cstripe_ are shown in Fig. 5d, e, respectively.

In the inset of Fig. 5c we plot 〈*X*〉 (T) versus |*T* − *T*_Cstripe_| at *H* = *H*_Cstripe_, and fit the data to obtain the critical exponents *a*_*T*stripe_ = 1.1 ± 0.2 and *n*_*T*stripe_ = 3.4 ± 0.6. We find same critical exponents for both *T* < *T*_Cstripe_ and *T* > *T*_Cstripe_ cases. The obtained values of critical exponents were used to collapse the data using Eq. (2). Figure 5c shows the collapse for all the distributions presented in Fig. 5a, b. Similar results are shown as a function of *H* for the case of* T* = *T*_Cstripe_ in the inset and main panel of Fig. 5f, where the critical exponents were found to be *a*_*H*stripe_ = 1.1 ± 0.3 and *n*_*H*stripe_ = 3.73 ± 1.0. (The detailed analysis technique followed is given in Supplementary Notes 3, 4 and 5 and Fig. 5 of Supplementary information.)

**Fig. 6:**
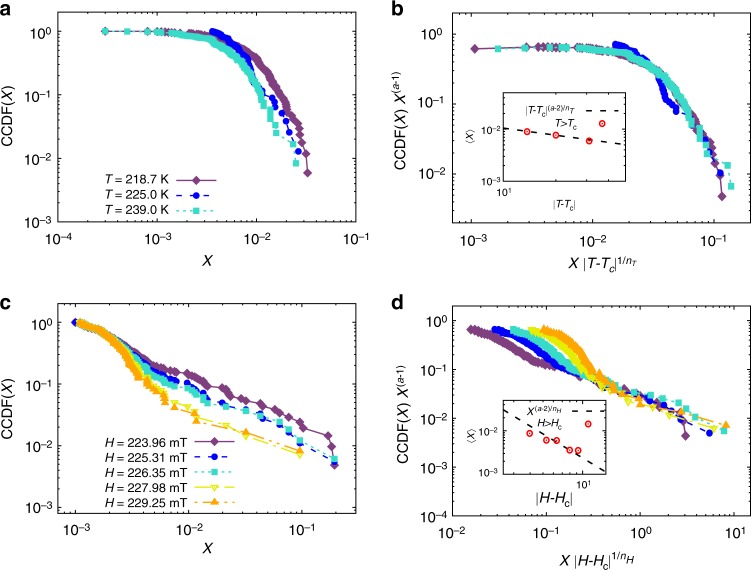
Scaling collapse for skyrmion phase: Complementary cumulative distribution function of domain cascades in the skyrmion phase, with critical values *T*_Cs*k*_ = 205.6 K and *H*_Csk_ = 220 mT. (**a**) Data at *H* = *H*_Csk_ and (**c**) Data at *T* = *T*_Csk_. Scaling collapse at *H* = *H*_Csk_ (**b**) and *T* = *T*_Csk_ (**d**). Inset: The average size of the jumps as a function of the distance to the critical point. The dashed line represents the fit to 〈*X*〉(*μ*) ∼ |*μ* − *μ*_C_|^(*a*−2)/*n*^, with *μ* = *T* or *μ* = *H*. The values of the critical exponents for field and temperature are *a*_*H*sk_ = 1.1 ± 0.2, *n*_*H*sk_ = 0.5 ± 0.2 and *a*_*T*sk_ = 1.1 ± 0.3, *n*_*T*sk_ = 2.06 ± 0.5, respectively

A similar analysis was performed for the skyrmion phase (shown in Fig. 6). We found that the critical exponents that collapsed the data best at *H* = *H*_Csk_ = 220 mT are *a*_Tsk_ = 1.1 ± 0.3 and *n*_Tsk_ = 2.06 ± 0.5, while at *T* = 205*K* ∼ *T*_Csk_, we obtained the critical exponents values of *a*_*H*sk_ = 1.1 ± 0.2 and *n*_*H*sk_ = 0.5 ± 0.2.

## Discussion

One of our important findings from jump characteristics is that once the skyrmion phase sets in there is hardly any evidence of sudden change in the periodicity, orientation, or number density of the distorted hexagonal lattice as is the case in stripe phase. Previous experimental studies have not reported the exact nature of changes occurring in the skyrmion phase relative to the size, morphology, or number density. It is likely that the topological protection that allows skyrmions to move easily through the lattice is also the reason that domain walls are not pinned and consequently show smaller magnitude of jumps.

Our analysis clearly shows critical behavior for both stripes and skyrmion phase that is centered around different critical field and temperature. The critical exponent *a* has a similar value for both phases, but exponent *n*_*H*_ takes different value for stripes compared to skyrmions. The similarity of the exponent *a* suggests that near the critical point the distribution of avalanche sizes for both stripes and skyrmions follows roughly the same power law distribution although the magnitudes are much different. Since long-range dipolar interactions play important role in the Fe/Gd heterostructure^22^, it is not surprising that the exponent *a* is similar in both cases. It is reported that dipolar interaction with frustration lead to small power law exponents *a* in other avalanche systems: for example, the Sherrington–Kirkpatrick avalanche model gives an exponent of *a* = 1 ± 0.1, which is very close to our findings^35^.

The difference in the *n*_*T*_ and *n*_*H*_ exponents between stripes and skyrmion, on the other hand, suggests that the divergence of the correlation length is much faster in one case than the other. Since all the critical exponents are not same for both the phases, this indicates that stripes and skyrmions belong to different universality classes. Apart from average size of the jumps, which is a non-universal quantity, we can therefore use the avalanche statistics and universal exponents *n*_*H*_ for distinguishing stripes and skyrmions.

Distinctly separate critical fields *H*_C_ and temperatures *T*_C_ indicates differences in energetics govern the formation of stripes and skyrmions. It is well established that stripe and skyrmion phases have different symmetry and dimensionality. One of the reasons for differences in jump size and critical exponents could be attributed to differences in dimensionality. A stripe is a one-dimensional structure whereas a skyrmion is a two-dimensional lattice. Results in Fig. 4 suggest that under an applied magnetic field, stripe domains undergo avalanches while skyrmions become locally distorted. Since we know the skyrmion winding number needs to be conserved, which makes them move relatively easily through lattice, the domain walls are weakly pinned leading to a small magnitude of jumps. As a consequence, topology and dimensionality influence the dynamic behavior of stripe and skyrmion phases.

Apart from field-induced domain fluctuations, thermal fluctuations are most likely enhanced near the critical temperature. Future studies will be directed towards understanding the nature of the disorder–order transition particularly that involves meron and skyrmion spin textures^36^. Indeed, study of fluctuations as a function of time will enable us to determine “correlation function” that may shed light into formation of skyrmions. It will be important to perform similar studies on a Dzyaloshinskii–Moriya-based skyrmion system and evaluate the critical nature of the helical and skyrmion state. Beyond magnetic systems, our X-ray-based technique can be applied to a wide variety of materials system, such as liquid crystals, polymers, ferroelectrics to directly study stochasticity and scaling behavior at the nanometer length scale.

## Methods

### Sample fabrication

The samples studied were nominally [Fe (0.34 nm)/Gd (0.4 nm)] × 80 multilayers deposited on Si_3_N_4_ membranes using DC magnetron sputtering in an ultrahigh vacuum (UHV) environment under a 3 mTorr argon environment with a 20 nm Ta seed and capping layers.

### Coherent soft X-ray scattering

Resonant coherent X-ray scattering experiments were performed at the Coherent Soft X-ray Science beamline of the Advanced Light Source at Lawrence Berkeley National Laboratory. The beamline was designed to produce linearly polarized coherent soft X-rays. The resonant condition was achieved by tuning the energy of the incident beam to the Fe L_3_ edge (≈707 eV photon energy or 1.75 nm photon wavelength). Since at resonant condition the X-rays are sensitive to the magnetization along the beam propagation direction, we used a normal incidence transmission scattering geometry to enhance the magnetic contrast. A 10 μm pinhole was put in the path of the incident beam to establish transverse coherence in the beam. Typical transverse coherence length of the beamline is about 5 μm. The existence of Airy fringes and magnetic speckle pattern in the measured data (see Fig. 1) clearly demonstrate the coherence of the soft X-ray beam. A charge-coupled device detector placed 0.5 m down-stream of the sample was used to record the scattered intensity patterns as a function of magnetic field over multiple field cycles and repeated at different temperatures.

## Supplementary information

Supplementary Information

## Data Availability

The data that support the plots within this paper and other findings of this study are available from A. Singh (e-mail: arnabsingh21@gmail.com) and/or the corresponding author upon reasonable request.

## Code availability

Computer codes that were written to analyze the data is available from A. Singh (e-mail: arnabsingh21@gmail.com) and/or the corresponding author upon reasonable request.
