# Publisher Correction: Scaling of domain cascades in stripe and skyrmion phases

**DOI:** 10.1038/s41467-019-10314-w

**Published:** 2019-05-21

**Authors:** A. Singh, J. C. T. Lee, K. E. Avila, Y. Chen, S. A. Montoya, E. E. Fullerton, P. Fischer, K. A. Dahmen, S. D. Kevan, M. K. Sanyal, S. Roy

**Affiliations:** 1 Saha Institute of Nuclear Physics, HBNI, 1/AF Bidhannagar, Kolkata, West Bengal 700064 India; 2 Materials Sciences Division, Lawrence Berkeley National Lab, Berkeley, CA 94720 USA; 3 Advanced Light Source, Lawrence Berkeley National Lab, Berkeley, CA 94720 USA; 4 Department of Physics, University of Illinois Urbana-Champaign, Urbana, IL 61801 USA; 5 Center for Memory and Recording Research, University of California San Diego, La Jolla, CA 92093 USA; 6 Department of Physics, University of California, Santa Cruz, CA 95064 USA

**Keywords:** Magnetic properties and materials, Phase transitions and critical phenomena, Spintronics

Correction to: *Nature Communications* 10.1038/s41467-019-09934-z, published online 30 April 2019

The original version of this Article contained an error in Fig. 4d, in which the label of the region to the left of the white dashed lines incorrectly read ‘Order stripes’. The correct version states ‘Disorder stripes’. This has been corrected in both the PDF and HTML versions of the Article.

