# Supplementary material for: Scaling of domain cascades in stripe and skyrmion phases
Source: Nat Commun. 2019 Apr 30;10:1988. doi: 10.1038/s41467-019-09934-z (PMC6491444; doi:10.1038/s41467-019-09934-z)
Supplement: Supplementary file 1 — Supplementary Information [file 41467_2019_9934_MOESM1_ESM.pdf]

Supplementary Information

Scaling of domain cascades in stripe and skyrmion phases

A. Singh, J. C. T Lee, K. E. Avila, Y. Chen, S. A. Montoya, E. E. Fullerton, P. Fischer, K. A. Dahmen, S. D. Kevan, M. K. Sanyal, S. Roy

We provide here information about scattering geometry in Supplementary Note 1and the mathematical definition of pair-wise correlation coefficient in Supplementary Note 2. Simple simulations to calculate correlation coefficient is provided in Supplementary Note 3. A typical histogram of jump size distribution and complementary cumulative distribution function (CCDF) is discussed in Supplementary Note 4. Finally the determination of the critical exponents is explained in Supplementary Note 5.

Supplementary Note 1

We provide a schematic of real and reciprocal space representation of stripe and skyrmion lattice. Linear arrangement of stripes results in two intense diffraction spots. The skyrmion are arranged as hexagonal lattice in real space that manifests as six-fold diffraction pattern.

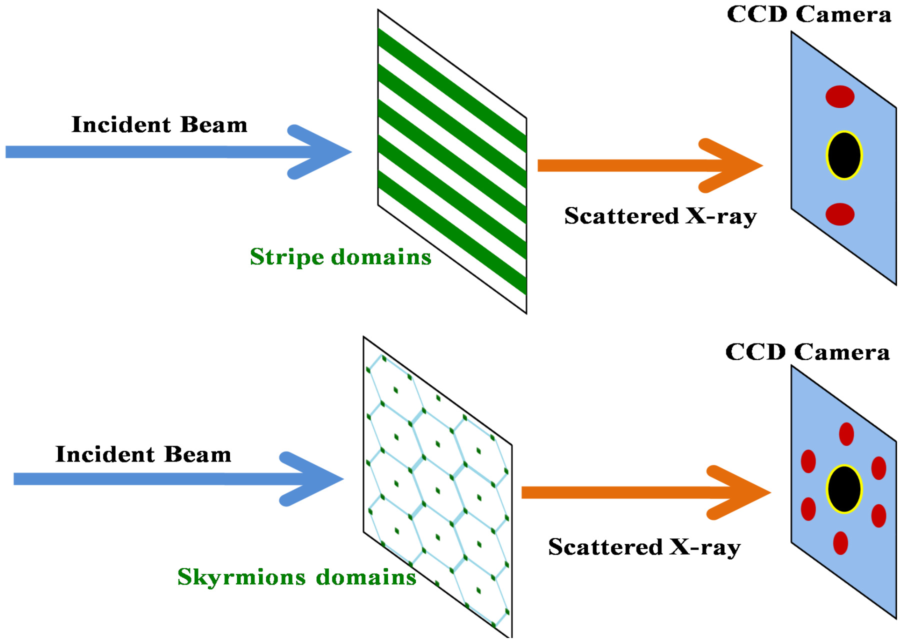

Supplementary Figure 1: Real and reciprocal space representation of stripes and skyrmions : Expected diffraction patterns on the detector from stripe and skyrmion domains. Red spots denote the magnetic diffraction peaks. The central black dot indicates direct-beam stopper.

Supplementary Note 2

Pairwise correlation coefficient (p) is calculated using the formula:

$$p = \frac{\sum_m \sum_n (A_{mn} - \bar{A})(B_{mn} - \bar{B})}{\sqrt{(\sum_m \sum_n (A_{mn} - \bar{A})^2)}\sqrt{(\sum_m \sum_n (B_{mn} - \bar{B})^2)}} \qquad (1)$$

where, A_{mn}= Intensity value at m^{th} row and n^{th} column of the first image (A), $\bar{A}$ = Average intensity value of the first image (A). B_{mn} and $\bar{B}$ are defined same way for the second image (B).

The value of p is 1 for correlated and 0 for uncorrelated images A and B. For two partially correlated images we get $0 < p < 1$.

Supplementary Note 3

We have carried out simple simulations to calculate correlation (PCC) from a model having two magnetic domains of rectangular shape in real space (see figure below). Each yellow rectangular box has an intensity normalized to one while the blue background has zero intensity. Then we performed Fourier transform (FT) of the 2D real space image and generated the 2D diffraction pattern in the reciprocal space. The reciprocal image was formed within a region of 1024 by 1024 pixels. By varying the position and size of the domains independently through random number generation we have created the diffraction pattern for 100 different images. Further, we also created a set of real space images by changing the centre of the diffraction pattern, which corresponds to a change in the lattice parameter. We then generated FT of those real space images. We selected a small region about the centre of the diffraction pattern and calculated the pair-wise correlation coefficient between two such regions of consecutive images with variation to size, position and centre separately for 100 images. The generated correlation plots resemble the PCC plots obtained from the experimental data.

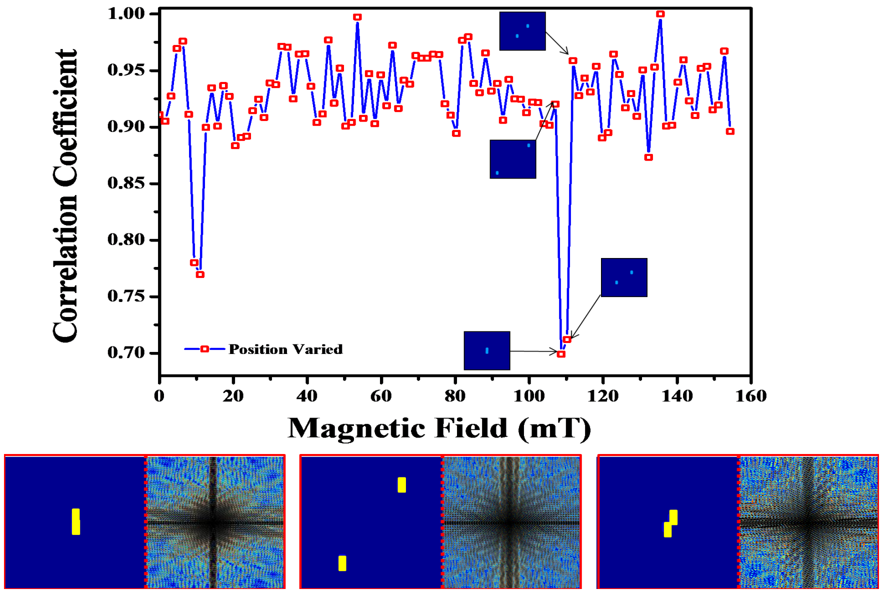

Supplementary Figure 2: Simulation of the effect of domain position changes: Plot of Correlation coefficient (PCC) between two consecutive diffraction images with variation to position. The x-axis corresponds to the image number which is assumed to vary due to applied magnetic field (1.575mT/image). The images below show the real space images and the corresponding diffraction pattern. The correlation coefficient plots show the presence of sharp jumps.

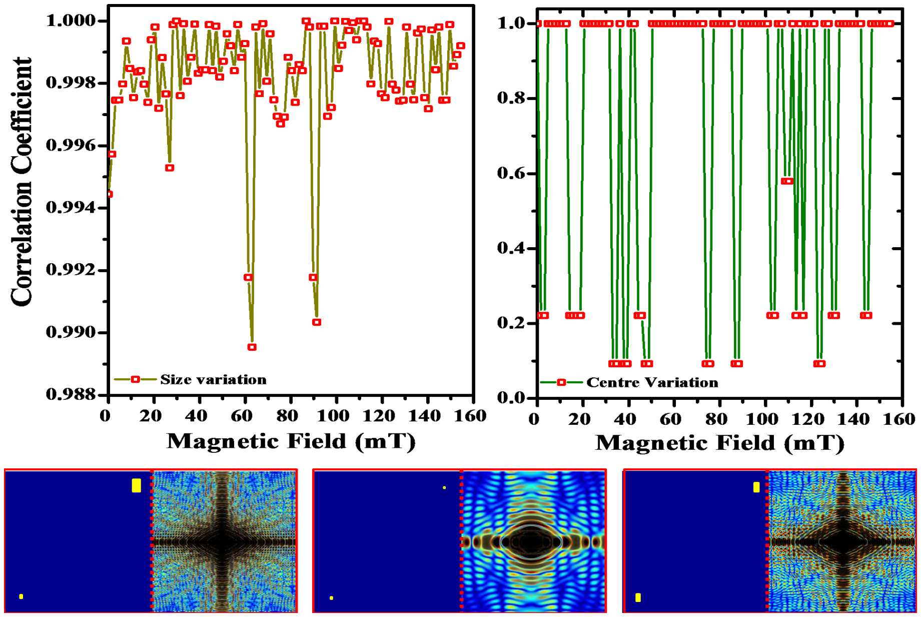

Supplementary Figure 3: **Simulation of the effect of domain size change:** Plot of Correlation coefficient between two consecutive diffraction images with variation to size and lattice parameter. The x-axis corresponds to the image number which is assumed to vary due to applied magnetic field (1.575mT/image). The real space images with the corresponding diffraction patterns are shown below.

Supplementary Note 4

Distribution of the Jumps as a function of its size at 300 K is shown Supplementary Fig 4 (a). The number of Jumps is extracted from the pair-wise correlation plots from over 100 field cycles. From the distributions we have calculated the cumulative distribution function (CDF) and the complementary cumulative distribution function (CCDF), defined as:

$$P_n = \frac{C_n}{\sum_{n=1}^{N} C_n} \qquad (2)$$

$$\mathrm{CDF} = \sum_{n=1}^{N} P_n \qquad (3)$$

$$\mathrm{CCDF} = 1 - \mathrm{CDF} \qquad (4)$$

where, C_n= Number of counts at the nth bin, N= total number of bins in the histogram and P_n is probability distribution function of the nth bin. In order to make the scaling function independent of the bin size and to minimise the effect of fluctuations at low counts we have done the statistical analysis using the CCDF.

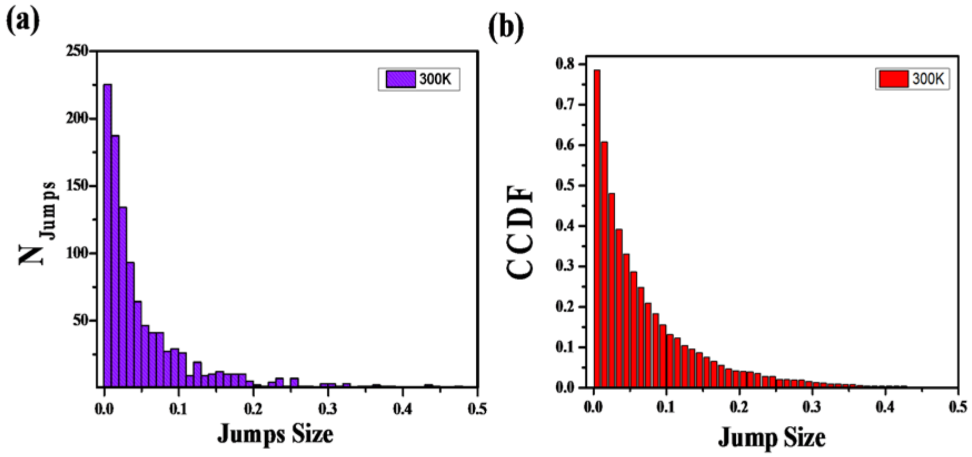

Supplementary Figure 4: **Distribution of jumps at 300 K:** (a) Histogram showing the number of Jumps as a function of Jump size. (b) Histogram of CCDF as a function of Jump size.

Supplementary Note 5

To determine critical exponents, the average value of the jumps and jumps squared was determined. First, we used the equations below to fit our data to obtain the critical exponents '*a*' and '*n*'. After that, we used the obtained critical exponents to collapse the data as shown in Figure 5 and Figure 6 in the manuscript for stripes and skyrmions, respectively.

$$\frac{\langle X^2 \rangle}{\langle X \rangle} \propto \Delta^{-1/n} \qquad (5)$$

$$\langle X^2 \rangle \propto \Delta^{(a-3)/n} \qquad (6)$$

$$\langle X \rangle \propto \Delta^{(a-2)/n} \qquad (7)$$

where,

$\Delta = |T - T_C|$, or, $\Delta = |H - H_C|$

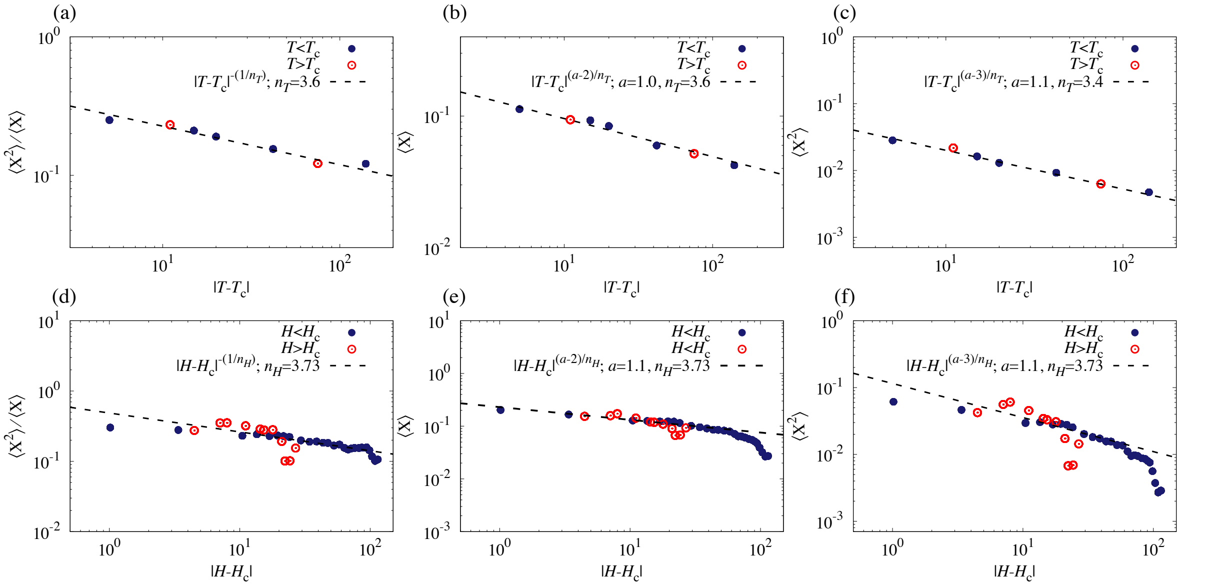

Supplementary Figure 5: **Extraction of critical exponents:** The plots shown above are for the stripe phase only. The first row corresponds to the critical field for temperatures below and above the critical value for (a) $\frac{\langle X^2 \rangle}{\langle X \rangle}$, (b) $\langle X \rangle$, and (c) $\langle X^2 \rangle$, respectively. The critical temperature for fields below and above the critical value is shown for (d) $\frac{\langle X^2 \rangle}{\langle X \rangle}$, (e) $\langle X \rangle$, and (f) $\langle X^2 \rangle$, respectively. Similar procedures were also followed for the skyrmion phase.
